# Current Insights into Nutritional Management of Phenylketonuria: An Update for Children and Adolescents

**DOI:** 10.3390/children12020199

**Published:** 2025-02-07

**Authors:** Dana-Teodora Anton-Păduraru, Felicia Trofin, Adina Chis, Lucia Maria Sur, Violeta Streangă, Dana Elena Mîndru, Olivia Simona Dorneanu, Diana Păduraru, Eduard Vasile Nastase, Romana Vulturar

**Affiliations:** 1 Department of Mother and Child Medicine, “Grigore T. Popa” University of Medicine and Pharmacy, 700115 Iasi, Romania; dana.anton@umfiasi.ro (D.-T.A.-P.); violeta.streanga@umfiasi.ro (V.S.); mindru.dana@umfiasi.ro (D.E.M.); 2 “Sf. Maria” Children Emergency Hospital, 700309 Iasi, Romania; 3 Department of Preventive Medicine and Interdisciplinarity-Microbiology, “Grigore T. Popa” University of Medicine and Pharmacy, 700115 Iasi, Romania; olivia.dorneanu@umfiasi.ro; 4 Discipline of Cell and Molecular Biology, Department 2-Molecular Sciences, “Iuliu Hatieganu” University of Medicine and Pharmacy, 400347 Cluj-Napoca, Romania; adinachis82@gmail.com (A.C.); romanavulturar@gmail.com (R.V.); 5 Department of Child and Mother, “Iuliu Hatieganu” University of Medicine and Pharmacy, 400347 Cluj-Napoca, Romania; sur.maria@umfcluj.ro; 6 Clinical Hospital of Infectious Diseases “Sf. Parascheva”, 700116 Iasi, Romania; eduard-vasile.nastase@umfiasi.ro; 7 “Dr. C.I. Parhon” Clinical Hospital, 700503 Iasi, Romania; diana_paduraru@email.umfiasi.ro; 8 Department of Internal Medicine II—Infectious Diseases, “Grigore T. Popa” University of Medicine and Pharmacy, 700115 Iasi, Romania

**Keywords:** phenylketonuria, nutrition, diet, breastfeeding, microbiota, children, adolescents

## Abstract

Considering the fact that even today in the 21st century, diet is the basis of the treatment in phenylketonuria (PKU), this review aims to provide a comprehensive analysis of existing current data from the last 15 years on dietary treatment and its impact on nutritional status and quality of life to identify gaps in knowledge and offer insights into future directions for optimizing nutritional care in PKU. Dietary treatment for PKU has evolved over the years, and in order to optimize and standardize the care, European PKU experts developed guidelines useful for both professionals and patients and their parents. The current literature underscores the essential role of diet in both managing PKU and preventing obesity, but malnutrition in these children is a complex issue that necessitates a multifaceted approach. The literature emphasizes the crucial role of dietary adherence in managing PKU. Advancements in therapy offer the potential to reduce the challenges associated with dietary phenylalanine (Phe) restrictions. Maintaining adequate levels of essential nutrients in children with PKU and monitoring trace element intake and micronutrient levels are vital for preventing deficiencies and ensuring optimal growth and development. Overall, the literature highlights the importance of personalized treatment strategies. Conclusions. Effective management of PKU necessitates strict dietary control and personalized treatment to maintain optimal blood Phe levels. Continuous monito-ring, nutritional education, and adherence to dietary recommendations are critical components in achieving the best patient outcomes. Future studies should also explore innovative therapeutic modalities, including gene therapy and novel dietary strategies that consider the gut–brain axis, to enhance the quality of life and mental health for individuals with PKU.

## 1. Introduction

Phenylketonuria (PKU) is an autosomal recessive disorder caused by a deficiency in the enzyme phenylalanine hydroxylase (PAH). This deficiency leads to the accumulation of phenylalanine (Phe) in blood and the brain, resulting in developmental delays, microcephaly, autism, neurocognitive impairment, psychiatric disorders, seizures, and, if untreated, severe and irreversible neurological damage [1]. The implementation of newborn screening (NBS) has enabled early diagnosis and treatment, ideally within the first 10 days of life for infants with Phe levels exceeding 360 µmol/L (6 mg/dL) [2,3,4].

The prevalence of PKU varies globally, with reported rates of 1 in 10,000 live births in the United Kingdom, 1 in 4000 in Italy, 1 in 4545 in Ireland, 1 in 11,200 in Finland, and between 1 in 16,300 to 1 in 34,500 in Brazil [2,5,6]. In Romania, prevalence estimates range between 1 in 7000 and 1 in 10,000 live births; the evaluation of the results from the entire country in 2013 was 1 case in 10,000 births [7].

PKU severity ranges from mild hyperphenylalaninemia (HPA) to classic PKU (the severe type of the disease), with a correlation between biochemical phenotype and genotype [8]. Classical PKU is characterized by Phe levels exceeding 1200 µmol/L (20 mg/dL) and poses significant risks to both patients (obesity, cardiovascular complications, neurodevelopmental disorders such as autism) and their pregnancies (growth retardation, microcephaly, intellectual disabilities, birth defects, including congenital heart defects) [9,10,11,12,13]. In these cases, genotyping is essential for identifying specific PAH gene mutations, clarifying the defect, and guiding treatment decisions. This genetic analysis also helps in assessing potential responses to therapies like tetrahydrobiopterin (BH4) and informs management strategies for affected infants. While genotyping can validate the mutations in the PAH gene, enhancing the understanding of the disorder’s background, it is not routinely conducted for every patient [14]. Nonclassical PKU that involves milder enzyme deficiencies or variations in the metabolic pathway can also lead to varying degrees of neurological impairment and developmental issues, necessitating careful management throughout the patient’s life [15]. Pregnant women with nonclassical PKU face heightened risks, including teratogenic effects on the developing fetus [16].

Cardiovascular complications in PKU arise from metabolic and dietary factors. The Phe-restricted diet often increases carbohydrate and fat intake, disrupting lipid metabolism and raising cardiovascular risk. Elevated Phe and low tyrosine (Tyr), crucial for vascular and neurotransmitter function, may also impair vascular health, especially in poorly managed PKU. Both diet and inherent metabolic imbalances contribute to these risks [17].

NBS has significantly altered the classification and management of patients with PKU, particularly those previously categorized as classical PKU. The early detection of elevated Phe levels allows for timely dietary interventions, which can prevent the severe neurological consequences associated with untreated PKU. A review by Blau et al. (2010) found that when treatment begins shortly after birth, the risk of cognitive impairment is significantly reduced, leading to a re-evaluation of the severity of the condition in these patients [18]. MacDonald et al. (2011) support the notion that early dietary management can effectively normalize Phe levels, suggesting that many patients may not fit the classical PKU profile if treated promptly [19]. van Wegberg et al. (2017), in their meta-analysis, concluded that the implementation of NBS programs has led to a significant decrease in the incidence of severe neurological outcomes in PKU patients [10].

Advanced screening methods, based on tandem mass spectrometry techniques, allow for accurate and efficient analysis of dried blood samples, facilitating early detection and treatment. By identifying high levels of Phe shortly after birth, healthcare providers can implement dietary interventions that are significantly reducing the risk of neurological damage associated with PKU, and, as mentioned, genetic analysis helps in identifying the molecular defect and also in assessing potential responses to therapies like BH4.

Maintaining plasma Phe levels within a therapeutic range of 120–360 µmol/L (2–6 mg/dL) is crucial to prevent intellectual disabilities and achieve positive patient outcomes. Despite advancements in treatment, dietary management remains the primary approach, even 90 years after PKU’s discovery. European guidelines recommend continuing dietary treatment until age 12 for patients with Phe levels between 360 and 600 µmol/L (6–10 mg/dL) as long as levels remain below 600 µmol/L (10 mg/dL). For patients with Phe levels above 600 µmol/L, treatment should continue beyond 18 years, though adherence into adulthood presents challenges, such as;

- A low-Phe diet supplemented with Phe-free protein substitutes, which must be strictly observed;
- Restriction of Phe intake and insurance of adequate nutrition from non-Phe l-amino acids and essential micronutrients;
- Monotony due to the limited variety of foods available;
- The abnormal taste that can diminish appetite for other essential foods, decreasing adherence to the diet;
- Neophobia;
- Variable bioavailability, sometimes the patients not having access to all the food, highlighting the necessity for ongoing monitoring and follow-up [20,21,22].

Effective management of PKU requires a multidisciplinary team, including a pediatrician, nutritionist, neurologist, psychologist, geneticist, general practitioner, family medicine physician, internist for those over 18 years of age, and laboratory medicine specialist. Additionally, effective management of PKU in female patients may involve an informed obstetrician.

### Motivation

The motivation behind this review stems from the critical need to address the complex dietary management challenges posed by this metabolic disorder. PKU requires lifelong adherence to strict dietary regimens to prevent cognitive impairments and other health complications, making nutrition a cornerstone of care. Despite advances in treatment, many children and adolescents with PKU face difficulties maintaining optimal Phe levels, resulting in nutritional deficiencies, suboptimal growth, and long-term health issues.

Given the evolving landscape of PKU management, including new therapeutic approaches and protein substitutes, it is essential to review current evidence on nutritional strategies, supplementation needs, and the impact of diet on physical and cognitive development. This review aims to provide a comprehensive analysis of existing research and current data on dietary treatment and its impact on nutritional status and quality of life, identify gaps in knowledge, and offer insights into future directions for optimizing nutritional care in this vulnerable population. In addition, we will include the topic of gut microbiome—an increasingly relevant topic in various conditions—as well as its relationship with emerging pharmacological therapies.

It also seeks to emphasize the importance of personalized dietary interventions and the role of education in supporting long-term adherence, thus improving outcomes and quality of life for children and adolescents with PKU.

## 2. Literature Search

### Search Strategy

This review analyzed 161 studies, initially identified through a search for the keywords “nutrition in phenylketonuria” or “diet in phenylketonuria”. Searches were carefully structured using targeted keywords and Boolean operators like “and”, “or” and “in” to improve accuracy. The choice of “nutrition” over narrower terms like “nutritional aspects” or “nutritional management” aimed to capture a broader and more diverse range of studies, ensuring a comprehensive review that addresses the multifaceted nature of PKU dietary needs for children and adolescents. It also acknowledges that relevant research may cover biochemical, dietary, clinical, and behavioral dimensions of nutrition in PKU beyond strictly defined management strategies.

Searches were conducted using established databases such as PubMed, Google Scholar, Cochrane, and EMBASE. In selecting the studies, we did not consider their type to avoid excluding any potentially relevant information they might provide. After gathering initial records, duplicates were removed. The first exclusion criterion was based on language; therefore, only articles written in English or Romanian were selected. Some articles were excluded after title screening due to misalignment with the review’s objectives. While title screening can be an efficient method to reduce the workload in a systematic review, it comes with the risk of prematurely excluding relevant studies. The choice to exclude at this stage should be made cautiously, ideally with clear criteria and independent reviewers to mitigate the potential downsides. For complex or interdisciplinary topics, it may be wiser to lean towards a more inclusive approach during title screening and defer exclusion decisions until after the abstract has been reviewed.

In selecting articles related to the impact of diet in PKU, priority was given to works published in the last 15 years, with a particular focus on studies from the past 5 years. This approach allowed for the inclusion of the most recent findings, supporting an up-to-date understanding of the interactions between diet, PKU, and general health as framed by established medical practices.

This extended timeline has been instrumental in highlighting shifts in research paradigms, while also providing historical context for recent advancements. Recent studies (published after 2018) have specifically examined interventions such as the use of glycomacropeptides (GMPs)-based medical foods or prebiotic and probiotic supplements, which could help mitigate the restrictive effects of the PKU diet.

During abstract screening, more articles were excluded based on criteria like relevance, date, accessibility, and research focus. Full-text reviews further eliminated studies due to issues of relevance, methodology, scope, quality, credibility, or language.

Chosen studies underwent a qualitative review by evaluating factors such as scrutinizing adherence to principles of scholarly composition, clarity, sample size, provision of pertinent data, articulation of results, and formulation of conclusions. For our review focused on providing current insights, it was more important to prioritize recent publications rather than those with high citation counts. Additional relevant sources were identified through manual reference checks. This revision improves clarity and flow while maintaining the original meaning.

## 3. Optimizing Nutrition in PKU: Key Strategies for Effective Management

### 3.1. General Overview

PKU was identified in 1934 by Asbjörn Fölling, but the first treatment was developed by Horst Bickel in the early 1950s and published in the Lancet in 1953 [23,24]. Currently, dietary management remains the cornerstone of PKU treatment and represents a unique nutritional challenge. The dietary regimen includes restricting natural protein intake according to individual tolerance while providing minimal amounts of Phe necessary for tissue growth and repair; consuming foods with low protein content; supplementing with low-Phe or Phe-free protein substitutes containing non-essential amino acids, essential fatty acids, micronutrients (such as vitamins, minerals, and trace elements), or GMPs [2,4,8,17,20].

### 3.2. Feeding the Future: Essential Nutritional Strategies for Infants with PKU

The approach to nutrition for infants with PKU has evolved markedly over time. Initially, while the benefits of breastfeeding were well-recognized in healthy infants, it was discouraged in PKU cases due to the necessity for precise monitoring of Phe intake. However, advances in understanding the low Phe concentration in breast milk have led to a revised approach. Nowadays, a combination of breastfeeding and Phe-free formula is considered an acceptable and beneficial dietary strategy for infants with PKU [25]. This shift in perspective highlights the need to investigate the prevalence and duration of breastfeeding in these infants, as well as its impact on serum Phe levels and weight gain [26]. Given the low Phe content in human milk (46 mg/100 mL) and its optimal nutritional composition for infant growth and central nervous system development, breastfeeding is now recommended for infants with PKU [2].

Zuvadelli et al. (2022) investigated various nutritional strategies for PKU infants and found that the duration of breastfeeding was positively influenced when a pre-measured amount of Phe-free formula was administered before breastfeeding. This study suggests a specific method for integrating breastfeeding and formula feeding in PKU management [27].

The nutritional composition of Phe-free infant protein substitutes is critical for managing PKU. A review of these substitutes in Italy indicated significant variability in their macronutrient, micronutrient, and functional components. Considering the variances within the products, close attention to these is essential to ensure effective metabolic control of PKU, support proper growth, cognitive development, gut microbiota health, and immune system function, and mitigate the risk of non-communicable diseases [28].

For newborns and infants with Phe levels exceeding 1000 μmol/L (16.66 mg/dL), a temporary cessation of all protein sources for 2–3 days is recommended, followed by the administration of Phe-free amino acid formulas. Subsequently, human milk, or a standard infant formula in restricted amounts, can be reintroduced. It is advised that 50–80% of the total protein intake should come from protein substitutes that comply with the Commission Delegated Regulation (EU) (2016/28) guidelines [10,17].

The first commercially available hydrolyzed casein substitutes were made available in 1958 in the United States as Lofenalac, developed by Mead Johnson. This product, a critical step in dietary management, provided a vital source of nutrition for individuals with PKU, allowing them to adhere to a low-Phe diet while ensuring adequate protein intake [29]. By around 1970, Phe-free formulas based on amino acids emerged, with their composition gradually enhanced to include polyunsaturated fatty acids (PUFAs) and prebiotics [30,31].

Various categories of protein substitutes are recommended for different age groups, though many are noted for poor taste and high osmolality. Transitioning between substitutes may involve challenges related to the acceptability of the new product, including differences in taste, smell, and consistency, as well as adjustments in daily routine and changing nutritional needs as the child grows.

In cases where the absorption or utilization of amino acids from protein substitutes is inefficient, a higher total protein intake may be necessary, particularly for patients with classic PKU [24,30].

For infants with PKU under 6 months of age, Phe tolerance ranges from 45 to 55 mg/kg body weight/day to maintain plasma Phe levels between 120 and 360 μmol/L (2–6 mg/dL). Special Phe-free formulas should provide 0.8–1.3 g protein/kg body weight/day representing a median of 53% of total protein intake with limits between 31 and 57% and 57% of energy with limits between 40 and 66%, depending on the age [2,32,33].

Complementary feeding in patients with PKU represents the introduction of complementary foods typically around six months of age, aligning with general pediatric guidelines. For PKU patients, the selection of appropriate foods is critical to maintaining low Phe levels while ensuring adequate nutrition [34]. Research indicates that the timing and type of complementary foods can significantly influence metabolic control in PKU patients. A study by Cassidy et al. (2023) found that parents who received comprehensive dietary guidance were more successful in managing their child’s PKU through complementary feeding [35].

Araújo et al. (2016) highlighted the importance of understanding Phe content in plant proteins, noting their favorable amino acid profiles for specialized diets [36]. Dimina et al. (2022) emphasized the nutritional value of combining plant protein sources to achieve a complete amino acid profile [37]. Plant proteins often have lower Phe ratios (1 g protein = 20–40 mg Phe) compared to animal proteins (1 g protein = 50 mg Phe), supporting diverse diets for effective Phe management [17,38,39].

MacDonald et al. (1996) emphasize the importance of tracking Phe intake from all foods for accurate dietary management in PKU. Consequently, it is suggested that foods with ≤0.5 g of protein per 100 g, or fruits and vegetables with ≤75 mg Phe per 100 g, can be consumed freely without measurement. Additionally, foods containing ≤ 25 mg Phe per 100 g, such as sugar, sweets, and starches, can be consumed without precise measurement. However, MacDonald et al. (1996) have found that increasing natural protein by 50% from low Phe foods does not significantly affect blood Phe levels. They also demonstrated that the consumption of fruits and vegetables containing 51–75 mg Phe per 100 g, with the exception of potatoes, does not impact metabolic control due to the poor bioavailability of Phe and low digestibility of proteins from these foods [40]. Factors influencing the bioavailability of natural protein include eating habits, oral mastication, and food processing, while plant protein absorption may be reduced by fiber content, the hydrophobic nature of plant proteins, and anti-nutritional factors such as hemagglutinins, phytic acid, protease inhibitors, glucosinolates, and tannins [41].

After introducing complementary feeding, parents of PKU infants may face several challenges, including;

- Difficulties in calculating protein content from various foods;
- Fussy eating behaviors;
- Refusal of certain foods;
- Mistakenly providing inappropriate foods;
- Allergies and intolerances;
- The need to carry specific foods while traveling;
- The necessity of preparing daily meals at home [42].

Food neophobia is a significant problem among children with PKU, as in the study of Evans et al. (2015) [43]. The same author, in a study from 2017, concluded that food neophobia in children with PKU may be associated with fear of eating unfamiliar foods, which could contain a source of protein or aspartame [44]. Due to the multifaceted nature of neophobia, Krupa-Kotara et al. (2024) analyzed food neophobia and its relationship with eating habits, preferences, and eating patterns, concluding that food neophobia shows a stronger association with established eating patterns than with individual taste preferences, while in the study of Bugi et al. (2024), food neophobia appears to be more influenced by the patient’s age and the year of PKU diagnosis than from body mass index (BMI), the Phe level at the time of diagnosis, parental education level, or the patient’s [45,46]. Given the research of Bugi et al. (2024) regarding the implications of food neophobia on dietary patterns and mental health in PKU patients, it is important to address both dietary and psychological aspects in the management of disease, with the purpose of influencing children’s food acceptance and overall health [46]. According to MacDonald et al. (2012), introducing solid foods around six months of age in PKU infants may reduce food neophobia and improve acceptance [31].

The same authors, MacDonald et al. (2010), found that patients may experience increased hunger, potentially leading to non-compliance with dietary restrictions, while van Rijn et al. (2006) demonstrated that the consumption of some low-protein foods high in fiber can enhance feelings of fullness [47,48].

Additionally, difficulties can arise from parental anxiety or fear of change, loss of parental control, and the child’s attachment to the previous substitute. Issues such as large volume, poor tolerance (e.g., vomiting, diarrhea, constipation), and parental preferences for flavors may further complicate the transition to complementary feeding [49].

### 3.3. Tailoring Nutrition for Children and Adolescents with PKU: Strategies for Growth and Development

The dietary regimen for individuals with PKU and elevated blood Phe concentrations remains a contentious issue, particularly for those aged over 16 years. The literature indicates a need for individualized dietary management that considers both biochemical markers and the psychosocial well-being of patients. It is recommended to maintain blood Phe concentrations between 120 and 360 micromoles/L (2–6 mg/dL) up to 12 years of age and between 120 and 600 micromoles/L (2–10 mg/dL) thereafter. The North American recommendations advocated for stricter dietary control compared to European guidelines, leading to variability in clinical practice and patient outcomes. Research on nutrition for adolescents with PKU underscores the critical need to control blood Phe levels throughout life to achieve optimal neuropsychological outcomes [20].

For patients over the age of 3, a viable alternative to Phe-free protein substitutes is GMPs derived from cheese whey. These GMPs contain limited amounts of amino acids, which have slower absorption and less hepatic degradation, such as arginine, histidine, leucine, Tyr, and tryptophan, and offer numerous benefits including probiotic effects, anti-cariogenic properties, gastric acid inhibition, antimicrobial action, appetite control, anti-inflammatory and immune-modulatory effects, bone health improvements, increased short-chain fatty acid production, and reduced nausea. GMPs can also be administered in conjunction with LNAAs to enhance satiety and lower postprandial plasma ghrelin levels [2,8,50,51,52,53,54].

Different authors investigated advancements in food sciences for PKU management, specifically focusing on GMPs, the results being presented in Table 1.

Different research has increasingly focused on the role of LNAAs in managing PKU—Table 2.

Furthermore, Lammi et al. (2023) found that free amino acids (L-AAs) exacerbate oxidative and inflammatory conditions in colon carcinoma cells induced by hydrogen peroxide (H_2_O_2_) and lipopolysaccharides (LPS). This adverse effect was mitigated when L-AAs were combined with GMPs, providing new preclinical insights into the potential mechanisms of these products in the context of PKU treatment [65].

### 3.4. Uncovering Nutritional Gaps: Addressing Deficits in the PKU Diet

The diet for people with PKU often misses essential nutrients, which are vital for the development of the children. Micronutrient imbalances are a major concern for PKU patients, who are at increased risk for deficiencies in zinc, selenium, iron, vitamin B12, and folate [26]. These deficiencies are influenced by various factors, including the type of treatment, biological mechanisms, and adherence to dietary regimens (Table 3).

According to Wild et al. (2019), non-adherent PKU patients exhibit lower circulating concentrations of key metabolites such as Tyr and arginine, which are inversely correlated with Phe levels [80]. Lower plasma arginine levels can disrupt key biological functions due to arginine’s role as a precursor for nitric oxide (NO), which is vital for vascular health and immune function. Arginine deficiency impairs NO production, increasing risks of vascular dysfunction, inflammation, and immune suppression, particularly during childhood characterized by rapid growth and development. Additionally, arginine supports protein synthesis, the urea cycle, and amino acid metabolism. Its deficiency may lead to metabolic imbalances, poor wound healing, and reduced adaptive immunity [81,82]. Moreover, the protein synthesis could be affected by the imbalance of some amino acids (elevated Phe, decreased Tyr, and threonine). The interplay between these amino acid concentrations suggests that the deficiency of arginine may contribute to the metabolic dysregulation observed in PKU, with an impact on neurological development and health outcomes [83].

The deficiency of Tyr due to restricted Phe intake can exacerbate the neurological consequences of PKU and can have implications for growth and development. Pena et al. (2018) highlight the importance of Tyr, which is often deficient in PKU diets, as it plays a crucial role in neurotransmitter synthesis and overall metabolic health [84]. In the study of Bugi et al. (2024), Tyr levels were found to be lower in PKU patients, with implications for neurotransmitter synthesis and overall brain function [46]. Daly et al. (2019) suggest that dietary interventions may need to be adjusted to manage Phe levels and to optimize Tyr intake, the same thing being supported by the study of Matuszewska et al. (2024) [55,85]. These studies underscore the necessity for dietary adjustments to ensure adequate Tyr availability, particularly in the context of a Phe-restricted diet.

Acosta et al. (2004) also highlighted the challenge of achieving optimal nutrient status due to the restrictive nature of the PKU diet, which often relies on non-natural sources of nutrients, reinforcing the need for iron supplementation [86].

Lubina et al. (2023) conducted a study on Latvian PKU patients using food diaries, which revealed deficiencies in fat intake and excesses in iron, selenium, and zinc. These findings highlight the complexities of nutrient management in PKU patients and the necessity of regular nutritional evaluations [87]. Vitamin B12 levels in children with PKU are a critical aspect of their nutritional health. Hvas et al. (2006) found a high prevalence of vitamin B12 deficiency among adult PKU patients who followed a less restrictive protein diet. Despite normal folate levels, many patients showed early signs of vitamin B12 deficiency due to inadequate intake of vitamins B12 and B6. The study also noted a correlation between reduced vitamin B12 intake and lower serum cobalamin levels, underscoring the importance of dietary guidance and supplementation to prevent deficiencies [73].

Inadequate intake of pyridoxine (vitamin B6) and its active form, pyridoxal phosphate, has been shown to have significant implications for vitamin B12 metabolism in patients with PKU. Blau et al. (2010) found that those with low pyridoxine intake exhibited lower serum B12 concentrations, suggesting a potential link between B6 deficiency and impaired B12 metabolism [23]. On the other hand, Bokayeva et al. (2024) concluded that individuals with PKU under a specific diet can achieve a vitamin status comparable to that of healthy subjects [88].

In 2008, Porta et al. considered that osteoclasts involved in bone reabsorption play a role in bone destruction in PKU. Also, the authors considered that an imbalance between bone formation and bone reabsorption might explain the pathogenesis of bone loss in PKU [72]. The same author, in his study about the impact of metabolic control on bone quality in PKU, concluded that diet discontinuation is associated with bone loss, suggesting that poor compliance to a specific diet is the main determinant factor of bone damage [89]. But the study of Koura et al. (2011) indicated that the low BMD was independent of dietary compliance and plasma Phe levels, suggesting that other factors may affect the bone health in PKU [90]. However, Doulgeraki et al. (2014) mentioned that regular monitoring of BMD and adherence to dietary guidelines are essential for improving bone health outcomes in patients with PKU [91]. Regular BMD monitoring is recommended by European Guidelines for conditions associated with compromised bone health, such as PKU, osteoporosis, or other metabolic bone disorders. The aim is to detect early bone density loss, evaluate the effectiveness of interventions, and guide treatment adjustments. For individuals with PKU, it is essential to assess BMD periodically, especially in adolescence or adulthood, when bone density may decrease, and in cases of poor metabolic control of PKU; evaluate risk factors: these may include age, gender, history of fractures, calcium and vitamin D intake, and compliance with diet and medication; intervene early if changes in BMD are detected by dietary modifications, calcium and vitamin D supplementation, or other appropriate medical management [20].

This finding underscores the need for regular monitoring of BMD in PKU patients to prevent osteoporosis and related fractures, with the mention that bone mineral density may not be available in the youngest children. Conversely, in the study of Geiger et al. (2016), BMD was normal and linked to dietary calcium intake, suggesting that essential nutrients from medical food-based diets support adequate vitamin D levels and BMD in PKU children [71].

Crujeiras et al. (2015) conducted a multicenter study on patients with HPA, including those with PKU, and found deficiencies in selenium, along with increased folic acid levels [92]. Hanusch et al. (2024) investigated cognitive and bone health in well-treated PKU children, finding improved inhibitory control compared to controls and no significant differences in bone turnover markers [93].

The complex nature of nutrient absorption in PKU patients, it necessitates further research to prevent deficiencies and optimize nutritional status. Additionally, the results of studies regarding dietary nonadherence and the risk of the appearance of nutritional deficiencies are controversial. The study of Wild et al. (2019) indicates that nonadherent PKU patients exhibit lower levels of essential metabolites such as Tyr and arginine, which are inversely correlated with Phe levels, suggesting a risk of inadequate nutrient intake. Conversely, metabolomic profiling has identified urinary biomarkers linked to poor dietary adherence, raising concerns about the nutritional status of these patients. The variability of patients responses complicates the establishment of a standardized approach, underscoring the need for further investigation into the relationship between dietary adherence and nutritional deficiencies in PKU [80].

### 3.5. Navigating Lifelong Management of PKU

The management of PKU is significantly influenced by cultural and social factors, particularly concerning gender dynamics.

#### 3.5.1. Gender Influence

Ziegler et al. (2005) highlight that dietary adherence in PKU patients often varies by gender, with societal expectations and roles shaping dietary practices and health outcomes. Women, in particular, may face additional pressures related to family responsibilities and cultural norms that dictate food preparation and consumption, impacting their ability to adhere to strict dietary guidelines [94]. According to Iqbal (2023), gender-specific attitudes towards food and health can lead to disparities in dietary compliance. For instance, men may have more autonomy in food choices, while women often negotiate dietary restrictions within the family context, which can complicate adherence to PKU management protocols [95]. A European study by Pinto et al. (2024) revealed a decrease in blood Phe control with age, with females showing better metabolic control than males in certain age groups. This suggests that gender may influence dietary management outcomes [96]. These insights underscore the necessity for healthcare providers to consider gender and cultural factors when developing interventions for PKU patients.

#### 3.5.2. The Age

Compliance is generally higher among younger children whose diets are managed by their parents. In contrast, adherence often decreases during adolescence due to the diet’s impact on social interactions, which can lead to isolation, stigma, and reduced quality of life (QOL). Poor adherence can result in diminished metabolic control and an increased risk of cognitive issues [4].

#### 3.5.3. The Moment of Initiation of Diet

Additionally, children with PKU on diet therapy from birth showed lower IgE levels compared to those who started therapy later, suggesting that early initiation of diet therapy may help mitigate immune-related issues [97].

#### 3.5.4. Frequency of Monitoring

According to the European guidelines, it is advised to measure Phe levels weekly for children 0–1 year, fortnightly for 1–12 years, and monthly for those 12–18 years [10]. For patients under 12 years with more than 50% of Phe values outside the target range for six months, more frequent Phe monitoring, counseling, psychological support, social assistance, and possible hospitalization are advised [20]. The study of Pinto et al. (2024) also highlighted that more frequent blood Phe monitoring is associated with better metabolic control, underscoring the importance of regular monitoring to optimize dietary management. Overall, maintaining optimal Phe levels through dietary interventions is essential to prevent long-term neurological consequences of PKU. Additional research is needed to establish clear guidelines for managing Phe blood levels during adolescence to enhance metabolic control and neurocognitive outcomes in PKU patients [96].

#### 3.5.5. Compliance to Treatment

Recent studies have also examined the impact of dietary non-compliance on cognitive development in children with PKU, finding that fluctuations in peripheral Phe levels can affect neurological development [59,98]. As per the European guidelines, persistent out-of-range Phe levels over six months may classify the patient as non-compliant, necessitating safety measures and notification to social services [20].

Wiedemann et al. (2020) discuss the risks of discontinuing the PKU diet during childhood, noting cognitive and emotional issues such as loss of intelligence quotient (IQ), learning difficulties, anxiety, and personality disorders in adolescents and young adults. This highlights the necessity for ongoing dietary management throughout adolescence and adulthood [99].

#### 3.5.6. The Concentration of Phe

The management of PKU has traditionally focused on maintaining Phe concentrations under the nonhyperphenylalaninemic range of 50–120 µmol/L. However, emerging evidence suggests that even modest increases in Phe levels can adversely affect cognitive performance. Elevated blood Phe levels rapidly deplete intracellular Tyr and dopamine in neuronal models, suggesting that even isolated Phe elevation alone is sufficient to disrupt CNS processes [100]. Koletzko et al. (2009) emphasize the need for ongoing monitoring of blood Phe concentrations to tailor appropriate treatment strategies. For individuals with blood Phe levels exceeding 600 μmol/L (10 mg/dL), lifelong treatment is recommended, with specific target ranges for different age groups. The researchers also stress the necessity of scheduled follow-ups based on age, adherence to treatment, and clinical condition to ensure optimal health outcomes for children with PKU. This thorough approach is vital for improving the health of these patients [66].

The study of Huijbregts (2002) on 57 children with early and continuous PKU treatment demonstrated that those with plasma Phe levels exceeding 360 µmol/L exhibited significant deficits in sustained attention, cognitive interference inhibition, and overall performance consistency compared to controls [101]. Recently, Trepp et al. (2024) did not underscore the importance of maintaining stricter dietary controls, focusing on the lack of effect of a 4-week elevation of blood Phe on the parameters measured in adults [102]. Comparatively, Thomas et al. (2023) emphasized the significance of within-participant studies, revealing that lower Phe levels significantly enhance cognitive performance, thereby reinforcing the necessity of optimal Phe management across all age groups with PKU [103].

Non-compliance with the treatment regimen, whether due to a lack of adherence or availability of specific products, can lead to increased blood Phe levels, irritability, anxiety, and adverse clinical outcomes [2]. De Giorgi et al. (2023) conducted a systematic review linking metabolic control with neurocognitive functions during adolescence, finding a positive correlation between maintaining low average Phe levels and improved neurocognitive outcomes [104]. Lichter-Konecki and Vockley (2019) underscored the importance of maintaining Phe levels within the therapeutic range of 120–360 µmol/L (2–6 mg/dL) to prevent negative outcomes and discussed the role of cofactor therapy, particularly for milder forms of PKU [3]. Anastăsoaie et al. (2008) found that instable blood Phe levels throughout life are more critical for cognitive function than overall Phe exposure, highlighting the importance of stability of blood Phe levels and of consistent management for cognitive functioning in children with PKU [105].

Children with PKU must strictly adhere to a low-protein diet to effectively manage their condition. Pinto et al. (2024) assessed blood Phe control in PKU patients across Europe and Turkey, finding that blood Phe levels often worsen with age, underscoring the need for continuous monitoring and management. The literature emphasizes that maintaining stable Phe levels within the therapeutic range is essential for optimal outcomes [96].

Other factors that can influence diet adherence are mentioned in Table 4.

### 3.6. Impact of PKU Diet on Gut Microbiota: Unraveling the Effects of Special Dietary Management

The gut microbiome plays a vital role in how dietary interventions affect individuals with PKU, with factors such as genotype, age, and diet influencing microbial composition and overall health [9]. Research indicates that changes in the gut microbiota can impact cognitive and behavioral functions through the gut–brain axis (GBA). In PKU, where a restrictive diet is a primary treatment, significant alterations in the gut microbiota have been observed, potentially linked to central nervous system (CNS) dysfunction, particularly through metabolic pathways like tryptophan (Trp) and kynurenine (KYN). A study by Parolisi et al. (2023) found that PKU patients receiving Phe-free amino acid medical foods (AA-MF) show imbalances in the KYN pathway, leading to neurotoxic metabolite production associated with neurodegenerative and inflammatory diseases. This underscores the need to monitor cognitive and behavioral well-being in routine clinical management. Future research should investigate how Trp metabolism, mediated by the gut microbiota, affects cognitive and behavioral functions in inborn errors of metabolism (IEMs) like PKU [112].

Recent studies have explored how PKU therapies affect the intestinal microbiota, noting that dysbiosis could worsen the clinical phenotype of PKU patients—Table 5.

In individuals with PKU, the gut microbiota is affected by the specific dietary restrictions necessary for managing the condition. Such a restricted diet alters the nutrient composition and availability within the gut. The reduction in protein intake and dependence on specialized medical foods can modify the types of nutrients accessible for microbial growth and activity. Consequently, the limited variety in protein sources and overall nutrient composition may result in alterations in the diversity of the gut microbiota. Beneficial bacteria that depend on a more varied diet may diminish, while other bacterial species could become more prevalent [116,117,118].

The gut microbiota produces short-chain fatty acids (SCFAs) through the fermentation of dietary fibers, and dietary changes can impact SCFA production, which is essential for gut health and immune function. The restrictive nature of the PKU diet may contribute to microbial imbalance or dysbiosis, which is linked to various gastrointestinal disorders and can affect overall health. To mitigate the effects of dietary restrictions, individuals with PKU often use supplements to ensure adequate intake of essential nutrients. The interaction between these supplements and the gut microbiota can further influence microbial health. Long-term adherence to a PKU diet may have enduring effects on the composition and function of the gut microbiota, potentially impacting overall health and metabolic processes [114,119,120].

### 3.7. Dietary Influence on Nutritional Status

Diet is essential in managing PKU, particularly during pregnancy. Maternal nutrition significantly influences fetal growth and development [82]. PKU presents unique challenges in pregnancy due to increased protein requirements and the risk of metabolic decompensation, necessitating a multidisciplinary approach to manage dietary and pharmacological interventions. Adequate protein intake, often achieved through specialized medical foods and amino acid supplements, is crucial. Pregnant women with PKU must maintain blood Phe levels between 120 and 360 µmol/L (2–6 mg/dL) to prevent maternal PKU syndrome (MPKUS), which can lead to complications such as microcephaly and congenital heart defects. Nutritional support ensures fetal development and prevents malnutrition, while deficiencies in essential amino acids can cause congenital abnormalities. Regular monitoring of plasma amino acids, dietary adherence, and psychosocial support are vital for optimal maternal and fetal health [121].

Malnutrition remains a concern for children with PKU due to dietary restrictions; however, obesity has become increasingly common, particularly among females (7.8–32.6%) [122].

Obesity in individuals treated for PKU is primarily linked to treatment rather than the genetic condition itself. The Phe-restricted diet often relies on high-calorie, low-protein medical foods, leading to a carbohydrate- and fat-rich diet that predisposes to weight gain, especially when combined with inactivity. Although energy intake may meet recommendations, the diet’s macronutrient composition is often suboptimal, highlighting the need for personalized monitoring to manage weight and metabolic risks [122]. Poor adherence to this regimen further increases the risk, with non-compliant patients showing higher rates of obesity [12,123]. Genetic factors in PKU minimally influence obesity compared to dietary and lifestyle impacts.

Cases of obesity in PKU patients, often linked to non-compliance with dietary restrictions, were first reported in the 1970s and 1980s [124]. Factors contributing to excess weight in PKU patients also include sedentary lifestyle, psychological influences, social environment, and low socio-economic status [5,125].

Stevioside, a natural sweetener from Stevia rebaudiana, is a safe and low-toxicity alternative to sugar, suitable for PKU patients as well as individuals with diabetes or obesity [126]. Tailored dietary plans and consistent monitoring of therapy adherence are crucial for achieving favorable outcomes. Annual assessments should include measurements of weight, BMI, plasma amino acids, and key micronutrients to guide effective management [20].

The relationship between BCAAs, which include leucine, isoleucine, and valine, and the risk of becoming overweight in PKU has garnered attention due to the fact that the metabolism of Phe is impaired, leading to dietary restrictions that may influence BCAA levels and overall metabolic health. Tummolo et al. (2022) observed that higher muscle mass in post-pubertal PKU patients correlated significantly with increased Phe intake and therefore with diet compliance but did not find an increase in fat mass after puberty [127].

The factors related to diet that influence the nutritional status of children with PKU are presented in Table 6.

### 3.8. Feeding Well-Being: How PKU Diets Shape the Quality of Life for Children and Their Caregivers

Recent research has explored how a specific diet for PKU affects the QOL of children with this metabolic disorder. Gama et al. (2023) found significant positive changes in food patterns, behaviors, and burden of care in children with PKU and their families after 6 months on sapropterin treatment [139]. Bensi et al. (2022) found that GMP-based protein substitutes do not significantly impact QOL. Their data suggest that patients generally accept GMP-containing protein substitutes well, likely due to their improved palatability [140].

Iakovou et al. (2019) conducted a study involving 110 mothers of PKU children to assess maternal QOL and social discrimination based on their educational background and residence. The study found that mothers with only a primary school education and those living in large cities (with populations over 300,000) experienced the greatest QOL impact. Conversely, mothers with a university degree reported the lowest levels of social discrimination. Additionally, mothers in smaller towns faced the highest levels of social discrimination. This underscores the need for psychological support for mothers of PKU children, particularly for those at greater risk of QOL damage and social discrimination [141]. In a related study, Iakovou et al. (2020) evaluated the effects of psychological support on mothers’ QOL in relation to their educational background. Among 42 mothers who received weekly psychological support for a year, those with a university degree achieved the most significant improvement in symptoms and QOL. Mothers with high school education saw moderate improvements, while those with a primary school education experienced minimal reductions in symptoms. This highlights the importance of tailoring psychological support to the educational background of affected mothers to improve their QOL [142].

Weglage et al. (2000), in their comparative study between PKU patients and diabetic patients, showed that problems such as depression, anxiety, physical complaints, and social isolation were significantly elevated in both PKU and diabetic patients, supporting a psychological perspective for the development of behavioral and emotional problems in these patients [143].

The impact of diet on cognitive status in children with PKU is crucial for managing this disorder. Research indicates that cognitive deficits, learning difficulties, and emotional problems are common in individuals with PKU, emphasizing the need for early detection and intervention [144]. Elevated blood Phe levels are linked to the severity of these cognitive issues, making a Phe-restricted diet essential for managing PKU. Early diagnosis and adherence to this diet are vital to prevent brain damage and cognitive dysfunction, highlighting the critical role of diet in cognitive outcomes for children with PKU [145]. Ongoing screening and assessment are necessary to identify cognitive impairments, which can often go unnoticed. The proposed Uniform Assessment Method for PKU, incorporating various screening tools for adaptive behavior, executive function, and emotional well-being, offers a comprehensive approach to monitoring cognitive status across different age groups [144].

## 4. Synergy or Conflict? Exploring the Interaction Between Pharmacological Therapies and Diet in PKU Management

Sapropterin, FDA-approved in 2007 and for Europe approved in 2009, enhances residual PAH activity, lowering blood Phe by ~30% and increasing dietary tolerance by up to 100% in responsive patients. Studies highlight its role in improving Phe control and reducing caregiver burden, though not all patients respond to treatment [4,146,147]. Feldmann et al. (2024) found that sapropterin dihydrochloride significantly improved full-scale IQ in patients, particularly when blood Phe fluctuations increased, indicating that it may enhance Phe tolerance and support cognitive development in PKU individuals [59].

Pegvaliase, approved in 2019 in the EU, offers enzyme substitution therapy for adults with severe PKU and adolescents from 16 years of age. It lowers blood Phe, supports protein intake, and improves quality of life but requires careful dose adjustments and monitoring due to potential side effects like anaphylaxis and arthralgia [4,146,148,149,150].

Newer therapies, such as sepiapterin, show promise, with trials reporting significant Phe reductions compared to sapropterin. However, further research is needed to optimize treatment protocols and understand long-term impacts [151,152,153].

## 5. Discussion

PKU is a metabolic disorder that demands rigorous dietary management to avoid adverse health effects. Ongoing research and discussion focus on the nutritional aspects of PKU.

The conventional treatment for PKU involves a strict low-Phe diet, which has been linked to complications such as gut microbiota disruptions and nutritional deficiencies. Research indicates that dietary interventions for PKU patients can disrupt gut microbiota, emphasizing the need for personalized therapeutic and nutritional strategies to address microbial imbalances [8]. PKU diets, which restrict high-protein foods, reduce the availability of proteins and amino acids essential for beneficial gut bacteria, potentially leading to shifts in microbial diversity and abundance. These dietary changes can impact microbial metabolism, affecting the production of short-chain fatty acids and other metabolites critical for gut health. The restrictive nature of the PKU diet may increase the risk of dysbiosis, which can adversely affect digestion and overall health.

Supplementation, often required to meet the nutritional needs of PKU patients, may further influence gut microbiota, necessitating careful management. Understanding the interactions between PKU therapies, gut microbiota, and cognitive and behavioral functions is vital for optimizing treatment and improving outcomes. Investigating the relationship between diet, genotype, microbiota composition, tryptophan metabolism, and neurological functions in PKU could inform the development of personalized interventions to address dysbiosis and enhance patient care. Collaboration with healthcare professionals to monitor and manage gut health, including microbiota assessments, is essential for individuals on PKU diets.

Micronutrient imbalances pose a significant concern for PKU patients, especially when a substantial portion of their nutrients comes from non-natural sources. Micronutrient supplementation is critical for PKU patients undergoing dietary treatment, either through the addition of these nutrients to Phe-free L-amino acids or through separate supplementation [26]. Deficiencies in micronutrients can have clinical and biological implications, highlighting the need for proper supplementation in PKU dietary management. Factors such as the age at which treatment begins, the type of treatment, dietary adherence, and dietary practices are crucial in determining the prevalence of vitamin and mineral deficiencies in this population. Maintaining adequate levels of essential nutrients in children with PKU and monitoring trace element intake and micronutrient levels are vital for preventing deficiencies and ensuring optimal growth and development. Restrictive diets with insufficient intake of Phe-free L-amino acids can lead to deficiencies in vitamins (B_12_, D) and minerals (iron, zinc, selenium), particularly during growth periods. The absence of animal protein often results in vitamin B12 deficiency. Studies emphasize the need to supplement Phe-free L-amino acids with essential vitamins and minerals to prevent deficiencies. Regular monitoring of ferritin, hemoglobin, total serum homocysteine, zinc, selenium, and vitamin D levels is also necessary [154]. Additionally, omega-3 supplementation has shown promising results in improving neurological deficits and motor function in children with PKU, highlighting its potential to enhance neurological function and motor skills.

Managing weight in the context of rising obesity rates presents a significant challenge. The current literature underscores the essential role of diet in both managing PKU and preventing obesity. By exploring the metabolic pathways related to these conditions and investigating new therapeutic approaches, researchers can devise more effective strategies to tackle the intricate relationship between diet, metabolic disorders, and obesity. Malnutrition in children with PKU is a complex issue that necessitates a multifaceted approach. Addressing eating problems, social restrictions, and other contributing factors can lead to targeted interventions that enhance the nutritional status and overall health of these children [62]. Future research should focus on the long-term impact of malnutrition in PKU patients and develop effective nutritional strategies to improve their health outcomes. The metabolic implications of obesity in PKU are still not fully understood, highlighting the need for further investigation into the causes of obesity within this population. Monitoring and preventing overweight and obesity in PKU patients should be a key focus for healthcare teams.

Dietary treatment for PKU has evolved over the years, yet there is no universal agreement on best practices. Van Spronsen et al. (2017) provide European guidelines for optimizing PKU care, emphasizing that diet remains the cornerstone of treatment. They offer recommendations based on blood Phe levels with specific targets for different age groups and stress the necessity of lifelong management for individuals with elevated Phe levels. These guidelines outline minimum requirements for management and follow-up based on age and clinical status [20].

The guideline of the American College of Medical Genetics and Genomics (ACMG) makes the following points;

- The lifelong treatment for PAH deficiency for individuals with untreated Phe levels > 360 μmol/L;
- Individuals with lifelong Phe levels ≤ 360 μmol/L have better intellectual outcomes than those who do not;
- Achieving Phe levels ≤ 360 μmol/L before conception is strongly recommended to prevent pregnancy complications and negative outcomes for the offspring;
- Genetic testing for PAH variants is recommended at birth to confirm diagnosis and guide therapy [155].

Factors such as social support, positive attitudes toward treatment, and access to specialized foods can significantly affect adherence to the PKU diet. Reviews of research on special diets for PKU highlight several important studies that illuminate the management and treatment of this condition. Early initiation of a Phe-restricted diet has been linked to improved cognitive outcomes in children with PKU. However, adherence often declines, particularly after adolescence. Despite recommendations for lifelong treatment, adherence to dietary interventions can be inadequate, potentially leading to nutritional deficiencies and medical complications.

Advancements in PKU therapy offer the potential to reduce the challenges associated with dietary Phe restrictions for patients and their families. These developments suggest a need to update follow-up guidelines to address crucial issues, including promoting breastfeeding and complementary feeding up to 2 years of age, improving metabolic control, monitoring variability in blood Phe and Tyr levels, screening for nutritional biomarkers, conducting neurocognitive and psychological assessments, evaluating bone health, addressing compliance difficulties, and supporting adolescents transitioning to adulthood with PKU. These recommendations are aimed at enhancing the management and care of adolescents with PKU [20].

Overall, the literature highlights the importance of personalized treatment strategies, consistent monitoring of blood Phe levels, and continuous nutritional and clinical follow-up to achieve optimal outcomes. Advances in technology have led to the development of automated systems for the rapid diagnosis and monitoring of PKU, which can significantly improve patient management. These systems provide accurate and sensitive measurements of Phe levels, facilitating timely dietary adjustments. However, the reliance on such technologies also highlights the need for healthcare infrastructure and access to specialized care, which may not be available for all patients [156].

Further research is necessary to investigate the long-term effects of treatment on cognitive function and brain development in both pediatric and adult patients. The findings emphasize that a comprehensive approach to managing the nutritional needs of PKU patients is crucial, considering not only their quantitative nutritional intake but also the complex interactions affecting their micronutrient status. Addressing these issues will help healthcare providers better support the overall health and well-being of children with PKU.

Research into the nutritional aspects of PKU in pediatric and adolescent populations has advanced considerably; however, several critical research gaps remain. Addressing these gaps is essential for enhancing understanding and improving the management of PKU, which, in turn, could lead to better patient outcomes. Firstly, there is a dearth of longitudinal studies examining the long-term effects of PKU diets on growth, development, and overall health throughout adolescence and into adulthood. Further research is needed to elucidate how dietary restrictions impact physical and cognitive development over extended periods. Considering Koch et al.’s study (1999), according to which the late-diagnosed intellectually disabled persons with PKU could participate in society and were able to arrest the neurodegenerative course, we consider that studies are also necessary for this category of patients [157]. Secondly, while existing research has predominantly focused on the management of Phe levels, there is a significant need for comprehensive studies assessing the adequacy of PKU diets in meeting all essential nutritional requirements. Investigations should address potential deficiencies in vitamins, minerals, and other nutrients and examine their implications for health outcomes.

Additionally, a deeper understanding of factors influencing dietary adherence among children and adolescents with PKU and their families is warranted. Apart from the previously mentioned factors, another factor that could influence adherence is the difficulty in interpreting food labels to manage dietary restrictions effectively. According to Hall et al. (2022), many patients and parents of PKU patients have difficulties interpreting protein exchanges from food labels, such as the lack of clarity regarding whether protein content was based on cooked or uncooked weights [158]. In addition to dietary management, adherence to new possibilities of treatment poses another significant challenge for PKU patients. Thus, the effectiveness of GMPs can be limited by individual preferences and the availability of suitable products.

Research should also explore how adherence to dietary regimens affects quality of life and psychological well-being. The impact of communication methods on adherence among PKU patients could be another subject of study.

Recent advancements in Precision Nutrition, particularly through the application of machine learning, present an opportunity to tailor nutritional advice based on individual needs. Kirk et al. (2021) noted that machine learning can integrate complex features of dietary intake, which can be particularly beneficial for PKU patients who require precise nutritional information to manage their condition effectively. By standardizing the nutritional composition of PKU products, the physicians and nutritionists can develop personalized dietary plans that enhance adherence and improve health outcomes. Therefore, the standardization of nutritional composition in PKU products not only fosters better communication among health professionals and patients but also aligns with emerging technologies in Precision Nutrition [159]. Other research has focused on novel therapeutics, one promising avenue involving the modulation of metabotropic glutamate (mGlu) receptors, particularly mGlu5 receptors. A study on Pahenu2 (ENU2) mice, which serve as a model for PKU, revealed increased mGlu5 receptor protein levels in specific brain regions, suggesting that targeting these receptors may ameliorate cognitive dysfunction associated with PKU [160]. Further research should focus on developing personalized nutrition plans that account for individual variations in metabolism, dietary preferences, and lifestyle. Studies are needed to assess how personalized approaches impact dietary adherence and health outcomes. Russo et al. (2024) emphasize the important role of food science and technology as a support for PKU dietary management, 3D food printing being an emerging technology for creating personalized, nutrient-optimized, and sensory-appealing foods for PKU patients, offering a new perspective in PKU management [161].

Moreover, understanding the psychosocial challenges associated with managing PKU diets is crucial, including the effects on social interactions, mental health, and family dynamics. Finally, evaluating the cost-effectiveness of various dietary strategies, medical foods, and supplements in PKU management could offer valuable insights for healthcare systems and families. Addressing these research gaps is vital for advancing management strategies and improving health outcomes for children and adolescents with PKU.

Our review on the nutritional management of PKU in children and adolescents faces limitations, including variations in study design, populations, and outcomes that complicate synthesis and reduce generalizability. Potential selection and publication biases may also skew findings. Furthermore, the review may lack robust evidence regarding the long-term effects of nutritional interventions on aspects like cardiovascular health or neurodevelopment, limiting its scope. These challenges can affect the overall comprehensiveness and applicability of the findings.

## 6. Conclusions

Effective management of PKU necessitates strict dietary control and personalized treatment to maintain optimal blood Phe levels, which are essential for growth and overall health. While emerging therapies such as sapropterin dihydrochloride and GMP-based protein substitutes show promise in enhancing Phe tolerance and cognitive outcomes, their nutritional impact must be thoroughly assessed to prevent adverse effects on weight and health. Continuous monitoring, nutritional education, and adherence to dietary recommendations are critical components in achieving the best patient outcomes. Further research is essential to evaluate the long-term effects and optimal dosing of pegvaliase, as well as the influence of breastfeeding, micronutrient supplementation, and Phe-free protein substitutes on young patients. Standardizing nutritional compositions for PKU products and developing tailored support programs for adolescents and young adults will be vital for improving adherence. Recent approaches have highlighted sepiapterin as a promising new therapeutic strategy for managing PKU due to its dual mechanism of action but also show potential in allowing patients to liberalize their restrictive diets while maintaining Phe control. Future studies should also explore innovative therapeutic modalities, including gene therapy and novel dietary strategies that consider the gut–brain axis, to enhance the quality of life and mental health for individuals with PKU.

Interdisciplinary approaches in developing novel treatments for PKU, combining pharmacological, computational, and machine learning strategies to improve patient outcomes and enhance the understanding of this complex disorder, are also necessary.

## Figures and Tables

**Table 1:** GMPs in the management of PKU.

Authors, Year	Results
Ney et al. (2009) [34] Daly et al. (2019) [55]	- GMPs containing aromatic amino acids are more palatable. - It is important to account for residual Phe (1.8 mg/g of protein equivalent) and Tyr in GMPs, subtracting these from the daily Phe allowance to prevent elevated blood Phe levels.
MacLeod et al. (2010) [56]	- Postprandial ghrelin was lowered after the consumption of GMP, being associated with fullness.
Ney et al. (2016) [51]	- GMPs are better accepted, with fewer adverse effects and proven safety
Singh et al. (2017) [57]	- GMP may influence satiety by its prebiotic properties.
Daly et al. (2020) [58]	- There was no indication to support the relationship between GMP and satiety.
Pena et al. (2021) [53]	- While GMP consumption did not significantly alter blood Phe levels, it did increase Tyr levels, with no changes noted in body composition or rates of overweight and obesity
Feldman et al. (2024) [59] Tosi et al. (2024) [60]	- GMP-based products varied in nutritional composition, often containing higher levels of sugars and saturated fats compared to standard amino acid mixtures. - Potential risk for developing overweight and obesity, particularly with ready-to-drink and bar formulations. - The improved palatability and nutritional quality of GMP-based products may enhance adherence to dietary therapy and potentially reduce obesity-related comorbidities

**Table 2:** The role of LNAAs in managing PKU.

	LNAAs’ Role		
Study	Lower Plasma and Brain Phe Levels	Alternative to the Traditionally Strict Phe-Restricted Diet	Increases Tyr Levels
Ney et al. (2014) [50]	X		
van Vliet et al. (2022) [61]	X		
Shyam et al. (2024) [62]		X	
Burlina et al. (2019) [63]			X
Schindeler et al. (2007) [64]	X	X	

Legend: X—highlight the elements identified in the study.

**Table 3:** Nutrient deficiencies in PKU patients.

Authors	Nutritional Gaps	Addressing Deficits	Post-Intervention Results	Follow-Up Outcomes
Koletzko et al. (2009) [66]	docosahexaenoic acid (DHA)	encapsulated fish oil	- improved central nervous system processing speed, motor function, and coordination	- DHA is critical for optimal neurological function in these patients, as the body’s endogenous conversion of alpha-linolenic acid may be inadequate
Jans et al. (2013) [67]	long-chain polyunsaturated fatty acid (LC-PUFA)	fish oil	- increased DHA levels - did not normalize arachidonic acid levels.	- indicating varied effects of omega-3 supplements on LC-PUFA status in PKU children
		KeyOmega (combination of DHA and arachidonic acid)	- achieved reference levels for both DHA and AA	
Van Spronsen et al. (2017) [20]	DHA	DHA supplementation	- enhancing CNS function and motor coordination	- faster visual evoked potential latencies and improved motor outcomes in PKU children
Wang et al. (2018) [68]	zinc	zinc supplementation	- role in supporting immune function	- defending against DNA viruses, further underscoring the significance of micronutrients in immune health
Robert et al. (2013) [26]	iron	ferrous sulfate supplementation	- significantly improved serum ferritin, transferrin saturation, and serum iron levels	- need for vigilant monitoring of iron levels in PKU patients - impact growth and development
	selenium	-	-	- selenium deficiency among PKU patients, along with deficiencies in other micronutrients such as zinc, iron, vitamin B12, and folate
Tanaka et al. (2018) [69]	calcium	calcium supplementation	inadequate intake of phosphorus and vitamin D	- concerns about bone health; - increase in bone formation, as evidenced by higher amplitude-dependent speed of sound values
Acosta et al. (1987) [70]	magnesium, potassium, and zinc	formula with higher levels of magnesium, potassium, zinc, and selenium	no significant changes in the mean concentrations of trace metals in urine, blood, or serum	- lower serum levels of zinc, selenium, and copper compared to children without PKU, suggesting potential deficiencies in these trace elements
	selenium		increase in selenium intake	selenium concentrations in the subjects remained lower than those in children without PKU
Geiger et al. (2016) [71]	vitamin D	-	-	- did not exhibit low serum vitamin D in children
Porta et al. (2008) [72]	serum Phe	-	osteoclastogenesis correlated with blood Phe concentrations	- elevated Phe levels may exacerbate bone resorption, contributing to bone loss
Hvas et al. (2006) [73]	vitamin B 12		- high prevalence of vitamin B12 deficiency - correlation between reduced vitamin B12 intake and lower serum cobalamin levels	- daily vitamin supplementation seems justified
Rojas-Agurto et al. (2023) [74]	- serum Phe - folic acid - vitamin B 12 - vitamin D		- serum folic acid was higher compared with controls - lower vitamin B 12 and D levels	- high plasma Phe levels to negatively impact both muscle and bone integrity
Keskin et al. (2023) [75]	- Fe, Ca, Zn, I, Se, vitamin B12 - long-chain polyunsaturated fatty acids	Phe-Free Protein Substitutes	efficacy and safety of new generation protein substitutes	- inadequate intakes in PKU patients who do not comply with treatment
Mendes et al. (2011) [76]	protein, calcium, phosphorus	Phe intake	affect bone age and mineral density	- non-adherence and nutritional inadequacies contributing to bone disease
Hargreaves et al. (2007) [77]	Coenzyme Q10 (CoQ10)		lowered CoQ_10_ level in PKU	- roles in oxidative stress and metabolic disturbances - the factor associated with the low CoQ10 level is the elevated Phe level
Sanayama et al. (2011) [78]	serum Phe		correlation between oxidative stress markers and serum Phe	- indicating that elevated Phe might aggravate oxidative damage and influence CoQ10 concentrations
Montero et al. (2019) [79]	CoQ10		an association between having low plasma CoQ values and being classic PKU patients	- plasma CoQ seems advi-sable to prevent the possibility of a chronic blood CoQ suboptimal status

**Table 4:** Factors that influence diet adherence.

Authors	Factors								
	1	2	3	4	5	6	7	8	9
Teruya et al. (2020) [106]	X	X	X	X		X	X	X	
Burlina et al. (2021) [4]				X		X			
Teruya et al. (2021) [107]		X						X	
Firman et al. (2022) [108]				X	X			X	
Pessoa et al. (2022) [109]			X						X
Pinto et al. (2023) [41]	X			X		X			X
Yagudina (2024) [110]	X			X		X	X	X	X
Beghini et al. (2024) [111]	X		X	X		X			

Table key: 1 = social/culture/psychological/behavioral/educational; 2 = the time; 3 = the cost; 4 = patient knowledge; 5 = age; 6 = healthcare professional support; 7 = poor social and/or family support; 8 = negative attitudes toward the diseases or dietary treatment; 9 = limited/insufficient availability of low-protein foods. X—Highlight the elements identified in the study

**Table 5:** Impact of PKU diet on gut microbiota.

Authors	Research Scope	Increase	Decrease	No Differences	Comments
Cox et al. (2013) [41]	Influence of diet high in plant proteins	*Bacteroidetes*	*Firmicutes*		
Zmora et al. (2019) [52]	Gut microbiota composition		*Firmicutes*		
Bassanini et al. (2019) [113]	Gut microbiota of children aged 4–18 years with PKU	*Blautia* *Clostridium* *Lachnospiraceae*	*Faecalibacterium*	*Methanobrevibacter smithii*	- *Faecalibacterium* = a marker of gut health influenced by carbohydrate quality; - Negative correlation between the glycemic index and *Faecalibacterium prausznitzii*, *Ruminococcaceae*, and *Roseburia*; - *Prevotella* was associated with higher fiber intake
Montanari et al. (2022) [114]	The effect of GMP supplementation on gut microbiota	*Agathobacter* spp. *Subdoligranulum*		Overall microbiota composition	- GMP improved calcium phosphate homeostasis, suggesting it could be a safe alternative protein source.
McWhorter et al. (2022) [115]	PKU group vs. Palynziq treated group	*Verrucomicrobia* *Lachnobacterium* genus vs. *Prevotella*			
Garcia-Gil et al. (2022) [2]	Influence of supplementation with galacto- and fructo-oligosaccharides on gut microbiota			Bifidobacteria	
Ubaldi et al. (2023) [8]	Evaluation of taxonomic groups and prebiotic supplementation	*Bifidobacterium* *Akkermansia*	*Firmicutes*/*Bacteroidetes* ratio		

**Table 6:** Influence of diet on nutritional status of children with PKU.

	Author, Year	Adequate Growth	Malnutrition	Weigh Gain/Obesity	Z Score	Comments
1	Acosta et al. (1998) [128]	X				- infants fed with Phe-free formula
2	Yilmaz et al. (2023) [2]	X				- infants fed with Phe-free formula - satisfactory blood Phe control, improved gastrointestinal symptoms
3	Singh et al. (2010) [129]	X			increase	- BH_4_ treatment improved dietary Phe tolerance
4	Kenneson and Singh (2021) [130]		X			- due to the limited intake of Phe-containing foods
5	Haitjema et al. (2022) [131]		X			
6	Bickel et al. 2001 [124]			X		- eating behaviors
7	Acosta et al. (2003) [86]			X		
8	van Rijn, M. et al. (2006) [48]			X		
9	Albersen et al. (2010) [132]			X		- higher body fat percentage
10	Demirkol et al. (2011) [133]				Normal	
11	Burrage et al. (2012) [134]			X		
12	Trefz et al. (2012) [135]			X		- imbalance in energy intake
13	Rocha et al. (2013) [136]			X		
14	MacDonald et al. (2008) [34]					- increased energy intake
15	Doulgeraki et al. (2014) [91]	X				- increase in fat mass, which correlated with increased blood Phe levels
16	Wang et al. (2020) [137]			X		- CTT peptide-endostatin mimic-kringle 5 fusion protein prevented weight gain and improved metabolic disturbances, increased energy expenditure and reduced obesity-related inflammation
17	Tummolo et al. (2022) [127]			X		- adherence to the PKU diet was associated with better body composition metrics, including total body water and muscle mass - the type of protein substitute influenced branch-chain amino acids levels, which could affect the risk of becoming overweight in adulthood
18	Dios-Fuentes et al. (2022) [138]	X		X		- individual variability within this population
19	Balci et al. (2024) [12]			X	Normal	
20	Tosi et al. (2024) [60]			X		- GMP-based protein substitutes have higher levels of sugars and saturated fatty acids, which may increase the risk of being overweight and obesity

Legend: X—Highlight the elements identified in the study.

## Abbreviations

AARP	CTT peptide-endostatin mimic-kringle 5, a specific fusion protein
AD-SoS	Amplitude-dependent speed of sound
BH_4_	Tetrahydrobiopterin
CNS	central nervous system (CNS)
DHA	Docosahexaenoic acid
GBA	Gut–brain axis
GMP	Glycomacropeptide
HPA	Hyperphenylalaninemia
IQ	Intelligence quotient
IGF-1	Insulin-like Growth Factor 1
Kyn	(Trp) and kynurenine
LNAA	Large neutral amino acids
LPS	Lipopolysaccharides
MPKUS	Maternal PKU syndrome
PAL	Phe ammonia lyase
Phe	Phenylalanine
PKU	Phenylketonuria
PUFAs	Polyunsaturated fatty acids
QOL	Quality of life
Trp	Tryptophan
Tyr	Tyrosine
